# Changes in organelle position and epithelial architecture associated with loss of CrebA

**DOI:** 10.1242/bio.201411205

**Published:** 2015-02-13

**Authors:** Rebecca M. Fox, Deborah J. Andrew

**Affiliations:** The Department of Cell Biology, The Johns Hopkins University School of Medicine, Baltimore, MD 21205, USA

**Keywords:** CrebA, Creb3-like, Drosophila, Salivary gland, Secretion, Secretory organelles, Septate junction

## Abstract

Drosophila CrebA facilitates high-level secretion by transcriptional upregulation of the protein components of the core secretory machinery. In *CrebA* mutant embryos, both salivary gland (SG) morphology and epidermal cuticle secretion are abnormal, phenotypes similar to those observed with mutations in core secretory pathway component genes. Here, we examine the cellular defects associated with *CrebA* loss in the SG epithelium. Apically localized secretory vesicles are smaller and less abundant, consistent with overall reductions in secretion. Unexpectedly, global mislocalization of cellular organelles and excess membrane accumulation in the septate junctions (SJs) are also observed. Whereas mutations in core secretory pathway genes lead to organelle localization defects similar to those of *CrebA* mutants, they have no effect on SJ-associated membrane. Mutations in tetraspanin genes, which are normally repressed by CrebA, have mild defects in SJ morphology that are rescued by simultaneous *CrebA* loss. Correspondingly, removal of several tetraspanins gives partial rescue of the *CrebA* SJ phenotype, supporting a role for tetraspanins in SJ organization.

## Introduction

Development of multicellular organisms requires the specialization of a myriad of cell types, each providing unique functional capabilities. Among the specializations of epithelial cells is the capacity to synthesize and secrete high levels of proteins and other substances. For example, the human pancreas secretes up to a liter of digestive enzymes daily (Levy et al., 2006) and the bovine mammary glands can produce six to seven gallons of milk per day during peak output (http://www.midwestdairy.com). The Drosophila salivary gland has proven an excellent model system for learning how epithelial organs achieve both the proper architecture and physiological adaptations for secretion (Chung et al., 2014). Indeed, studies of the Drosophila salivary gland identified CrebA as a direct transcriptional activator of genes encoding the core secretory machinery, including the protein complexes involved in targeting and translocation of nascent polypeptide chains into the ER, anterograde and retrograde trafficking of proteins within and between the ER and Golgi, as well as the elaborate post-translational modifications of proteins that occur within both organelles (Abrams and Andrew, 2005; Fox et al., 2010). Subsequent studies revealed that the five human CrebA bZip orthologs, known as the Creb-3-like (Creb3L) proteins, have similar activities, although loss of any one of the five mammalian orthologs has milder consequences than loss of the single Drosophila *CrebA* gene (Barbosa et al., 2013; Fox and Andrew, 2015; Fox et al., 2010).

Efficient secretion in epithelial cells requires a high degree of polarization, with bulk secretion within epithelial glands directed toward the apical (lumenal) surface (Hirano et al., 1991; Rousso et al., 2013; Schmidt et al., 2001; Viau et al., 1994). Epithelial polarity is manifested by the localized distribution of membrane and junctional proteins to unique domains within the plasma membrane (Knust, 2000; Martin-Belmonte and Mostov, 2008; Nelson et al., 2013; Tepass, 2012), polarization of microtubules (Kurihara and Uchida, 1987; Martin-Belmonte and Mostov, 2008; Meads and Schroer, 1995), as well as the localization of secretory vesicles just below the apical surface (Geron et al., 2013). A number of transmembrane proteins, including atypical cadherins (Chung and Andrew, 2014; D'Alterio et al., 2005; Schlichting et al., 2006), Zona Pellucida (ZP) proteins (Bökel et al., 2005; Fernandes et al., 2010; Jaźwińska et al., 2003), Drosophila Stranded-at-second (SAS) (Schonbaum et al., 1992) and others (Zhang and Ward, 2009), localize specifically to the apical surface, and appear to play a role in controlling apical membrane identity and size, likely through direct interactions with proteins on either side of the plasma membrane (Bökel et al., 2005; Chung and Andrew, 2014). Other transmembrane proteins, such as integrins and their associated complexes, preferentially localize to the basal membrane, serving to attach epithelial organs to an underlying basement membrane or basal lamina (Brown, 2000; De Arcangelis and Georges-Labouesse, 2000; Domínguez-Giménez et al., 2007; Marsden and DeSimone, 2003). The lateral surfaces of epithelial cells contain a number of unique junctional complexes that function to separate distinct membrane domains within cells, to attach neighboring cells, to provide rigidity and structure to the entire organ, to allow movement of small molecules from one cell to the next, and to limit diffusion of larger molecules from one epithelial surface to the other (Donato et al., 2009; Geiger et al., 1983; Guo et al., 2003; Knust, 2002; Koch and Nusrat, 2009; Lehmann et al., 2006; Nelson et al., 2010; Niessen, 2007; Wu et al., 2008; Yu and Yang, 2009).

Most junctional complexes are conserved between vertebrates and invertebrates, although the position of junctional complexes within the lateral domain differs slightly. Specifically, the junctional complexes that provide barrier function – tight junctions (TJs) in vertebrates and septate junctions (SJs) in invertebrates – are positioned differently with respect to the adherens junctions (AJs) (Willott et al., 1993; Woods and Bryant, 1993). Vertebrate TJs are located apical to the AJs, whereas invertebrate SJs are located just basal to the AJs. The major known protein constituents of both TJs and SJs are the four transmembrane span proteins known as claudins (Brandner, 2009; Tsukita et al., 2009). These proteins are thought to form interlocking extracellular domains that prevent diffusion of water and solutes. SJs have an additional “fencing” function, separating the apical from basolateral regions of the plasma membrane. Importantly, mutations in Drosophila SJ genes result in changes in the overall dimensions of epithelial organs – typically causing increases in either the length or width of the apical lumen (Behr et al., 2003; Wang et al., 2006; Wu and Beitel, 2004). The changes in epithelial organ dimensions observed with mutations in SJ genes are linked to defects in the polarized secretion and subsequent modification of an apically secreted extracellular matrix (Wang et al., 2006).

Recent studies have also revealed localization of some unexpected molecules to the SJs in insects. For example, Na^+^/K^+^ ATPase localizes to the SJs in Drosophila and mutations in the corresponding gene affect paracellular barrier function in much the same way as loss of other SJ proteins (Genova and Fehon, 2003; Paul et al., 2003). Molecules key to overall epithelial polarity also localize to SJs, including Discs Large, Lethal Giant Larvae and Scribble – proteins that counteract the activity of the apical determinant Crumbs (Crb) to establish and maintain overall cell polarity (Bilder and Perrimon, 2000; Strand et al., 1994; Woods and Bryant, 1991). Interestingly, loss of any single SJ component appears to disrupt the localization of most, if not all, of the others (Baumgartner et al., 1996; Lamb et al., 1998; Oshima and Fehon, 2011; Ward et al., 1998). This interdependence suggests that large macromolecular complexes contribute to SJ structure and function.

Here, we describe the cellular defects associated with the loss of the single Drosophila member of the Creb3L family, *CrebA*. Whereas many of the observed phenotypes are consistent with the loss of a transcription factor that coordinately upregulates nearly every component of the early secretory pathway, defects in the localization of multiple organelles, both secretory and non-secretory, are also observed. Intriguingly, there is significant membrane accumulation in the SJs of *CrebA* mutants, suggesting that SJs may have the additional role of providing a reservoir for excess plasma membrane. Finally, initial characterization of a class of genes whose transcription is upregulated in *CrebA* mutants – the tetraspanins – suggests that they may provide scaffolding function for SJs.

## Results

### Loss of CrebA alters organelle positioning

**Fig. 1.**
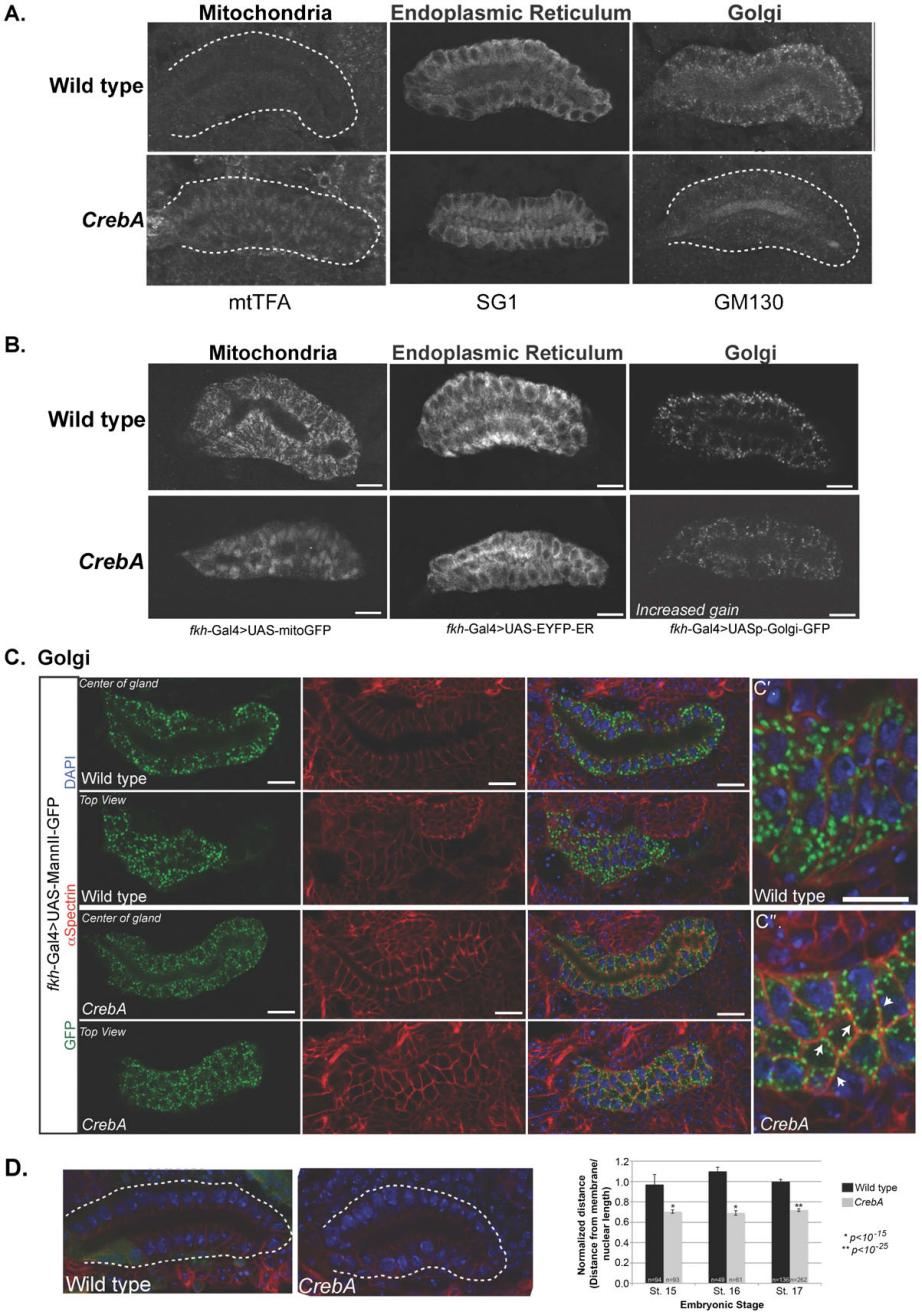
CrebA mutant salivary glands (SGs) display altered organelle localization. (A) Stage 15 embryos were fixed and stained with antibodies recognizing the mitochondria (mtTFA), endoplasmic reticulum (SG1) and Golgi (GM130). Note changes in localization of the mtTFA and SG1 staining intensity as well as reduced Golgi staining in the *CrebA* mutants SGs. White dashed lines outline the SG. (B) Fkh-Gal4 driving expression of mitochondrial (left panels), ER (middle panels) and Golgi (right panels) markers was examined in stage 15 WT (top panels) and CrebA mutant (bottom panels) SGs. (C) UAS-MannII-GFP (green) labels the Golgi stacks, which are distributed throughout the SG cells in both WT and *CrebA* mutants (left panels). α–Spec labels the lateral membranes (red in panels in right three columns) and DAPI labels the nuclei (blue in panels in right two columns). Confocal z-stack reveals that in wild-type SGs, the Golgi stacks are largely dispersed in the cytoplasm (C′). In *CrebA* mutants, the stacks are smaller and often found near the membrane (white arrows), as revealed by co-staining with α–Spec (C″). (D) Staining with α–Spec (red) and DAPI (blue) reveals that the nuclei are located closer to the basal surface in *CrebA* mutants. The white dotted line outlines the gland and GFP (green) labels the balancer chromosome marker used to identify heterozygous animals. Measurements of nuclear position in WT and *CrebA* mutant SGs (distance from the basal membrane to center of nucleus, divided by nuclear length) revealed a significant decrease in the distance from the basal membrane to the nuclei in *CrebA* mutants at embryonic stages 15–17. p-values were determined using a two-tailed Student's t-test. Error bars represent standard deviation. Scale bars: 10 µm.

The bZip transcription factor CrebA upregulates genes encoding the known protein components of the early secretory pathway in the salivary gland (SG) and other high capacity secretory organs (Abrams and Andrew, 2005; Fox et al., 2010). Previous studies revealed that loss of *CrebA* results in decreased apical secretion and minor morphological defects in the SGs; the SGs are slightly crooked compared to those of wild-type (WT) embryos (Andrew et al., 1997). To examine defects associated with *CrebA* loss at the cellular level, the abundance and distribution of proteins associated with secretory and non-secretory organelles were analyzed by confocal microscopy. Striking changes in organelle positioning were observed in *CrebA* mutants, especially with the mitochondria. Whereas in wild-type (WT) SG cells, mtTFA mitochondrial transcription factor staining was evenly distributed at very low levels throughout the cell, in *CrebA* mutant SGs, mtTFA staining was concentrated in a small subcellular domain, most often found in an apical region (Fig. 1A). In WT SGs, ER staining with antiserum to the SG specific ER protein prolyl-4-hydroxylase αSG1 (SG1) was most intense on the basal side of the cell; in *CrebA* mutants, however, SG1 staining was reduced overall, with the most intense signals near the apical surface (Fig. 1A). GM130 Golgi staining was significantly reduced in the SGs of *CrebA* mutants relative to WT (Fig. 1A).

To circumnavigate the transcriptional effects of *CrebA* loss on expression of secretory organelle proteins [levels of both *SG1* and *GM130* transcripts are reduced in *CrebA* mutants (Fox et al., 2010)] and to further examine changes in organelle positioning, the Gal4-UAS system was used to drive expression of organelle-specific GFP reporters using *fkh*-Gal4, an SG driver whose expression is unaffected by *CrebA* loss. *fkh*-Gal4 driven UAS-mito-GFP exhibited similar changes in localization as observed with the mtTFA marker; in *CrebA* mutants, staining was concentrated in a small domain, most often in the apical region of the cell (Fig. 1B). Similarly, whereas the UAS-EYFP-ER marker staining in WT SGs was more intense basally, *CrebA* mutant SGs had more intense staining in the apical side of the cell (Fig. 1B). For Golgi staining, two different UAS-GFP reporters were used: a GFP insertion tag on a galactosyl-tranferase – UASp-GFP.Golgi – and UAS-Mannosidase II (MannII)-GFP, an integral Golgi membrane protein. In WT SGs, both UASp.GFP.Golgi and UAS-MannII-GFP localized to puncta distributed throughout the cytoplasm (Fig. 1B,C). In *CrebA* mutants, the puncta were smaller and appeared more concentrated near the lateral plasma membrane (Fig. 1B,C). Confocal z-stacks of SGs co-stained with an antibody to αSpectrin (α–Spec), a marker for the lateral membranes, revealed that the UAS-MannII-GFP staining often localized adjacent to the α–Spec labeled membranes in *CrebA* mutant SGs (Fig. 1C). Finally, consistent with the more apical positioning of the ER and mitochondria, the nuclei were positioned more basally in *CrebA* mutants (Fig. 1D). Thus, every organelle examined, both secretory and non-secretory, showed changes in localization in the absence of *CrebA*.

### Overall cell polarity is not affected by loss of CrebA

**Fig. 2.**
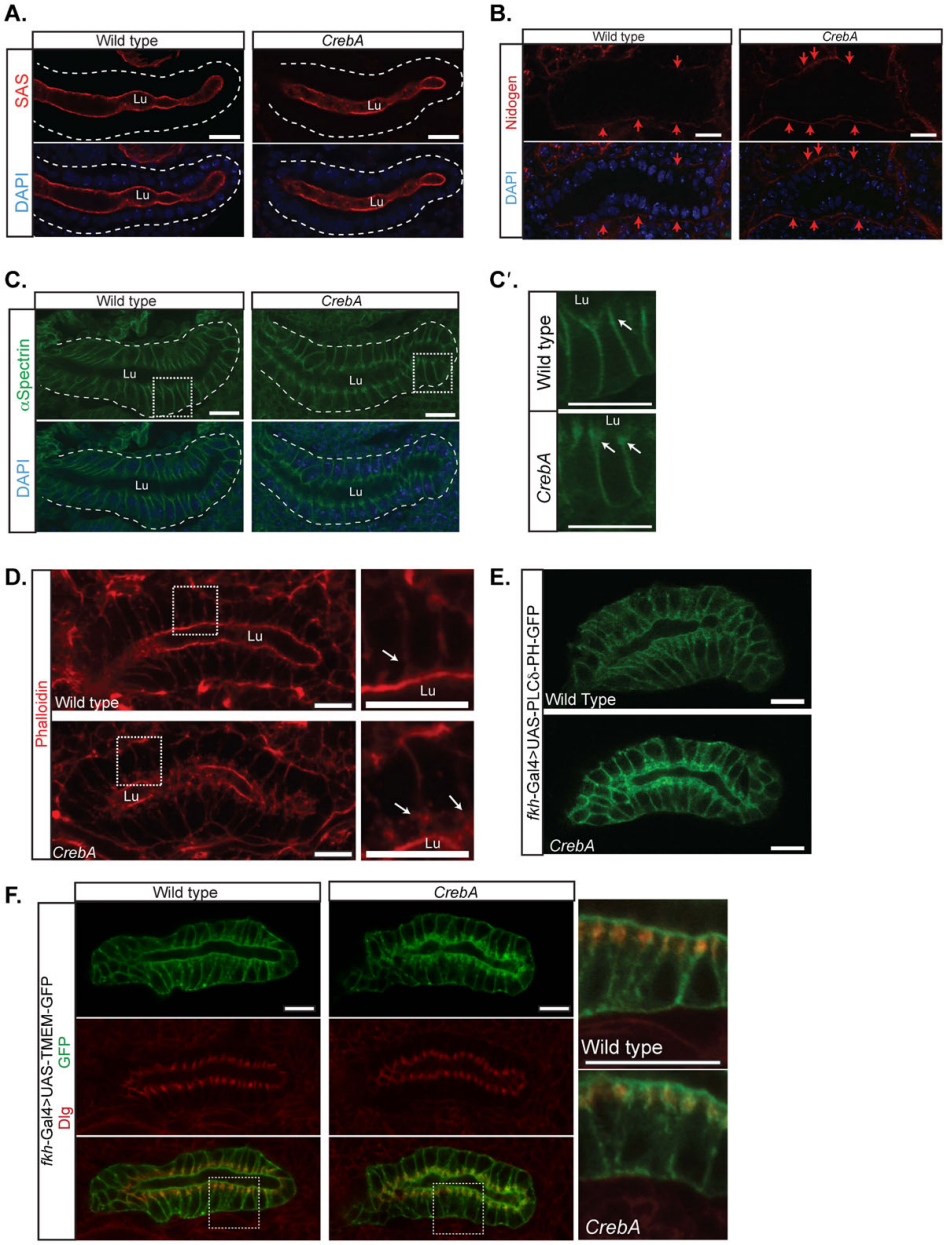
Overall cell polarity is unaffected in CrebA mutant SGs but SJs show additional membrane accumulation. (A) αSAS marks the apical surface of the WT (left panel) and *CrebA* mutant SGs (right panel). Dashed line shows outline of SG. (B) The SG basal ECM, visualized using the Ndg antibody, appears the same in WT (left panel) and *CrebA* mutants (right panel). (C) α–Spec staining labels the lateral membranes of WT SG cells (left panel). In *CrebA* mutants, staining also localizes to the lateral membrane (right panel) but there is increased accumulation of α–Spec just below the apical surface (arrows in higher magnification images to the right, C′). Dashed line shows outline of SG. (D) Phalloidin staining of WT reveals robust accumulation just beneath the apical surface, whereas phalloidin staining of *CrebA* mutants also reveals additional actin accumulation in lateral domains close to the apical surface (arrows). Boxed regions show regions magnified in right panels. (E) Staining with PLCδPH-GFP, a PtdIns(4,5)P2 sensor (von Stein et al., 2005), reveals accumulation along the apical and lateral surfaces in WT, with significant additional accumulation in the lateral domains close to the apical surface in *CrebA* mutants. (F) Co-staining with Tmem-GFP (green) and Dlg (red) reveals that the additional membrane accumulation in the lateral domain close to the apical surface of *CrebA* mutants corresponds to the septate junctions. Boxed regions show regions magnified in right panels. All SGS are embryonic stage 16. Scale bars: 10 µm. Lu, lumen.

To determine if the observed changes in organelle localization are due to changes in overall SG cell polarity, embryos were stained for the apical membrane markers SAS and Crb (Fig. 2A; data not shown), the basal extracellular matrix protein Nidogen (Ndg) (Fig. 2B) (Hynes and Zhao, 2000), and the lateral membrane protein α–Spec (Fig. 2C). None of the markers showed any change in distribution in *CrebA* mutants relative to WT except α–Spec (Fig. 2A–C). In *CrebA* mutants, normal lateral localization of α–Spec was observed but additional accumulation was seen in a lateral region just below the apical membrane (Fig. 2C, see below). Thus, despite the observed changes in organelle positioning in the SGs of *CrebA* mutants, overall cell polarity is unchanged.

### CrebA mutants have extra membrane and actin accumulation at the septate junction

To explore the increased α–Spec accumulation observed in the lateral regions of *CrebA* mutant SGs, embryos were stained for cytoskeletal proteins and for GFP reporters that mark the entire plasma membrane. In WT SGs, phalloidin (actin) staining was most intense along the apical surface, with much less intense staining along the lateral membranes. *CrebA* mutants exhibited a very similar staining pattern with the exception of increased actin accumulation along the lateral membrane, just below the apical surface (Fig. 2D). Tubulin accumulation was no different in WT versus *CrebA* mutant SGs (data not shown). *fkh*-Gal4 driven expression of two plasma membrane markers, UAS-PLCδ-GFP and UAS-TMEM-GFP, revealed increased accumulation of GFP-stained membrane in the lateral region just below the apical surface in *CrebA* mutants (Fig. 2E,F). To determine if this region corresponds to any of the epithelial junctions that localize along the lateral membrane, WT and *CrebA* mutant SGs expressing *fkh*-Gal4 driven UAS-TMEM-GFP were stained with markers for the adherens junctions (AJ) and the septate junctions (SJ). The increased GFP accumulation, which could be detected as early as embryonic stage 14, overlapped all SJ markers tested, including Coracle (Cora), NeurexinIV (NrxIV) and Discs large (Dlg), but did not overlap AJ markers (Fig. 2F, data not shown). Interestingly, although there was clearly an increase in SJ-associated membrane markers there was no overt increase in levels of any of the three SJ proteins examined.

### Electron microscopy confirms cellular changes in CrebA mutants

**Fig. 3.**
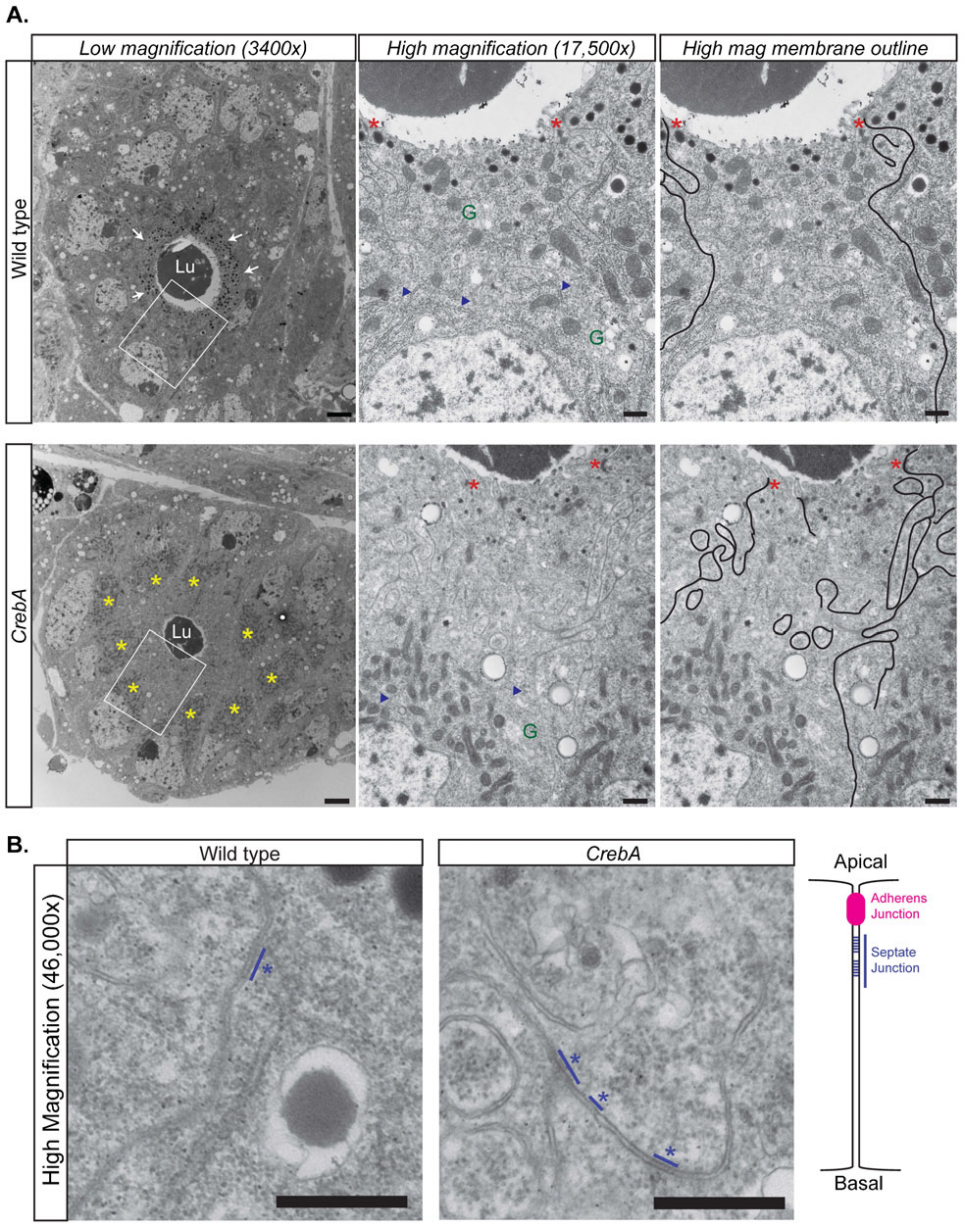
TEM reveals increased membrane at the SJ in *CrebA* mutants. (A, left panels) Cross-sections of stage 16 WT (top panels) and *CrebA* mutant SGs (bottom panels) showing reduced lumen size (Lu, lumen), decreased number of large secretory vesicles (white arrows) and the accumulation of mitochondria (yellow asterisks) apical to the nuclei in CrebA mutants. Regions in white boxes are magnified in the middle and right panels. (Middle and right panels) Higher magnification (17,500×) images of the apical region of a single cell revealing a significant increase in lateral membrane that is outlined in black in the right-most panels. Red asterisks mark AJs, blue arrowheads point out rER, ‘G’ indicates Golgi. (B) High magnification (46,000×) images of the lateral membranes from the apical-lateral region of both WT and CrebA mutant SGs reveal ladder-like structures typical of SJs (blue lines and asterisks). Schematic drawing on the right is showing the position of the SJ relative to the AJ along the lateral membrane. Scale bars: 2 µm (A, left panels); 0.5 µm (A, middle and right panels); 0.1 µm (B).

**Fig. 4.**
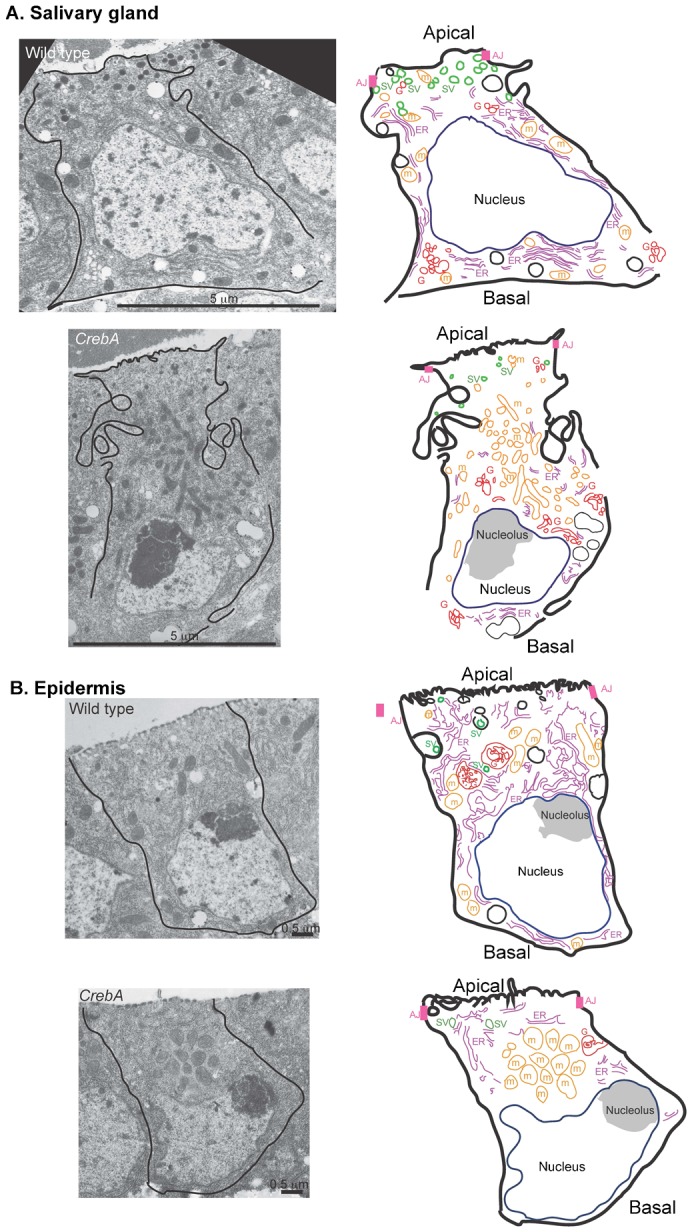
TEM analysis reveals changes in organelle positioning in both the SG and epidermis. (A) TEM images of single WT and *CrebA* mutant salivary gland cells (left), with cartoon drawings to the right that show the organization of the organelles in the micrograph of a single stage 16 wild type salivary gland cell to the left. (B) TEM images of single WT and *CrebA* mutant epidermal cells (left) with cartoon drawings to the right that show changes in organelle localization observed in a *CrebA* epidermal cell to the left. Scale bars: 5 µm (A); 0.5 µm (B).

To more closely examine the organelle and membrane phenotypes, TEM sections from both WT and *CrebA* mutant late embryonic SGs were examined. Previous TEM analysis revealed that *CrebA* mutants display phenotypes consistent with expected secretion defects, including reduced lumen size and fewer, smaller secretory vesicles (Fox et al., 2010). A more detailed examination of sagittal and cross sections revealed additional changes associated with loss of *CrebA* that are consistent with those observed with confocal imaging. Whereas in WT SG cells, the mitochondria were evenly distributed throughout the cell, the mitochondria of *CrebA* mutant SGs were concentrated in a region apical to the nuclei (Fig. 3A). Both a reduction in the amount of ribosome-studded ER (rER) membrane and a reduced number of Golgi-like structures were observed in *CrebA* mutants compared to WT (Fig. 4A). What rER could be seen in SG cells was mostly found apically and was near or within the mitochondrial clusters. Finally, whereas the lateral membranes of WT SG cells were generally linear, with a few minor bends near the apical surface, the lateral plasma membranes of *CrebA* mutant SG cells had a striking increase in the number of convolutions in this region (Fig. 3A). High magnification images of the regions containing membrane convolutions revealed stretches of ladder-like structures in the lipid bilayer, indicating that these membrane folds correspond to the septate junctions (Fig. 3B).

TEM analysis of the epidermal cells in *CrebA* mutant and WT embryos also revealed dramatic changes in organelle localization. Similar to the SG, mitochondria were evenly distributed throughout the WT epidermal cells and were clustered in a small apical region in *CrebA* mutant epidermal cells (Fig. 4B). *CrebA* mutant epidermal cells also had reduced ribosome-studded ER membranes that were in close proximity to the mitochondria, and secretory vesicles were both fewer and smaller than in WT. Also, consistent with the cuticle defects observed in first instar *CrebA* mutant larvae (Abrams and Andrew, 2005), structures at the apical surface were more irregular than in WT. Unlike our observation in the SG, no overt expansion of the apical region of the lateral membrane was evident in the *CrebA* mutant epidermal cells (Fig. 4B).

### SJ membrane accumulation is not linked to decreased secretory function

**Fig. 5.**
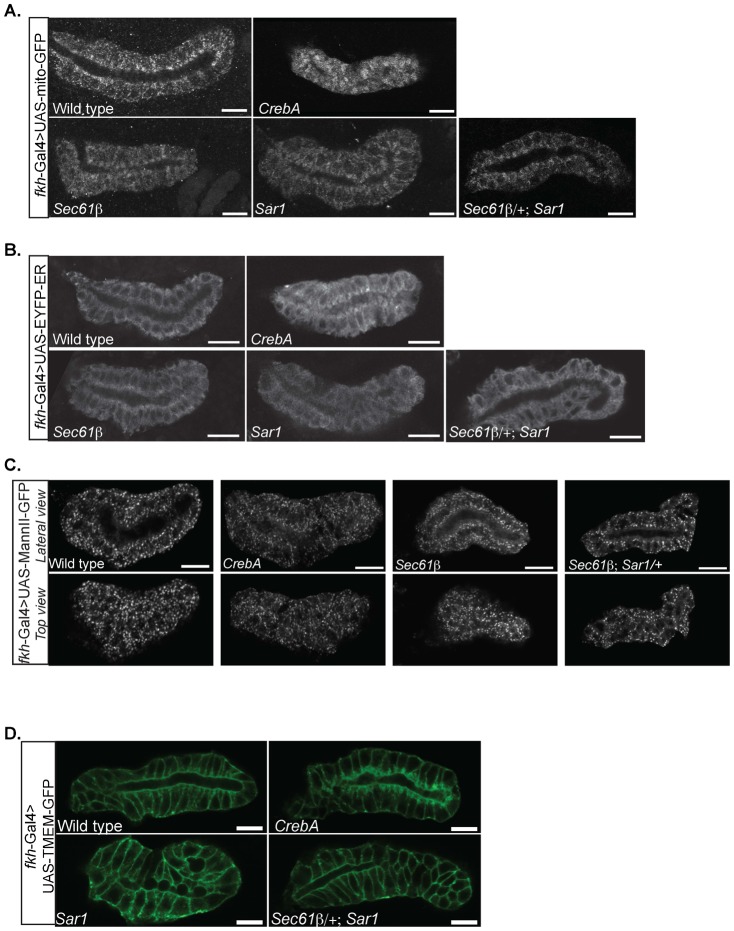
Secretory pathway component mutations do not affect SJ membrane accumulation but show mild changes in organelle distribution. (A) Mito-GFP staining reveals apical clustering of mitochondria in *CrebA* mutants that is also observed to some extent in *Sec61β* mutant SGs, in *Sar1* mutant SGs, and in *Sec61 β/+; Sar1* compound mutant SGs (top panels). (B) EYFP-ER staining reveals apical clustering of ER in *CrebA* mutants that is also observed to some degree in *Sec61β* mutants SGs, in *Sar1* mutant SGs, and in *Sec61 β/+; Sar1* compound mutant SGs (top panels). (C) MannII-GFP staining reveals fewer, smaller Golgi puncta in *CrebA* mutants that is also observed to some extent in *Sec61β* mutant SGs, in *Sar1* mutant SGs, and in *Sec61β ; Sar1/+* compound mutant SGs. Top panels show a slice through the lumen and bottom panels show a slice near the surface. (D) TMEM-GFP staining reveals expanded SJs in *CrebA* mutants that are not observed in WT SGs, in *Sar1* mutant SGs or *Sec61 β/+; Sar1* compound mutant SGs. Scale bars: 10 µm. Lu, lumen.

Both the excess SJ membrane phenotype and the organelle positioning defects could be either a consequence of the reduced secretory capacity in *CrebA* mutants or could be due to expression changes of other uncharacterized CrebA-dependent target genes more directly involved in cell organization. To address this question, P-element insertion alleles disrupting nine different components of the early secretory pathway were analyzed. The organelle positioning defects in SG cells from embryos homozygous for five of the single mutations showed mild changes in organelle positioning. *Sec61β* and *Sar1* mutants had the most overt phenotypes of the nine lines assayed, with both mutants showing some apical enrichment of mitochondria and ER (Fig. 5A,B). Moreover, both mutant lines showed slight reductions in signal intensity with the UAS-MannII-GFP reporter as well as staining more associated with the plasma membrane (Fig. 5C). Organelle positioning was also examined in embryos homozygous for either *Sec61β* or *Sar1* and heterozygous for the other mutation since very few embryos homozygous for both mutations developed. Salivary gland organelle relocation phenotypes were similar to those observed with the single mutants in *Sec61β* or *Sar1* (Fig. 5A–C). We conclude that a subset of the cellular changes observed in *CrebA* mutant SGs can be linked to reduced secretory function.

To ask if reduced secretory function is the basis for the increased SJ membrane, UAS-TMEM-GFP was expressed in the *Sar1* mutant as well as the *Sec61β/+; Sar1* compound mutant (Fig. 4D). Unlike the organelle positioning phenotype, changes in the lateral membrane were not observed in these mutants. This finding was confirmed by staining the *Sec61β; Sar1* mutants with antibodies for α–Spec and actin (data not shown). Thus, although the changes in organelle localization in *CrebA* mutants can be attributed to reduction in secretory function, the changes in SJ membrane are likely to be linked to expression changes of other CrebA target genes and/or are a consequence of the severe reduction in levels of nearly all of the proteins that normally populate the secretory organelles (Fox et al., 2010).

### Candidate CrebA target genes involved in SJ membrane accumulation

**Fig. 6.**
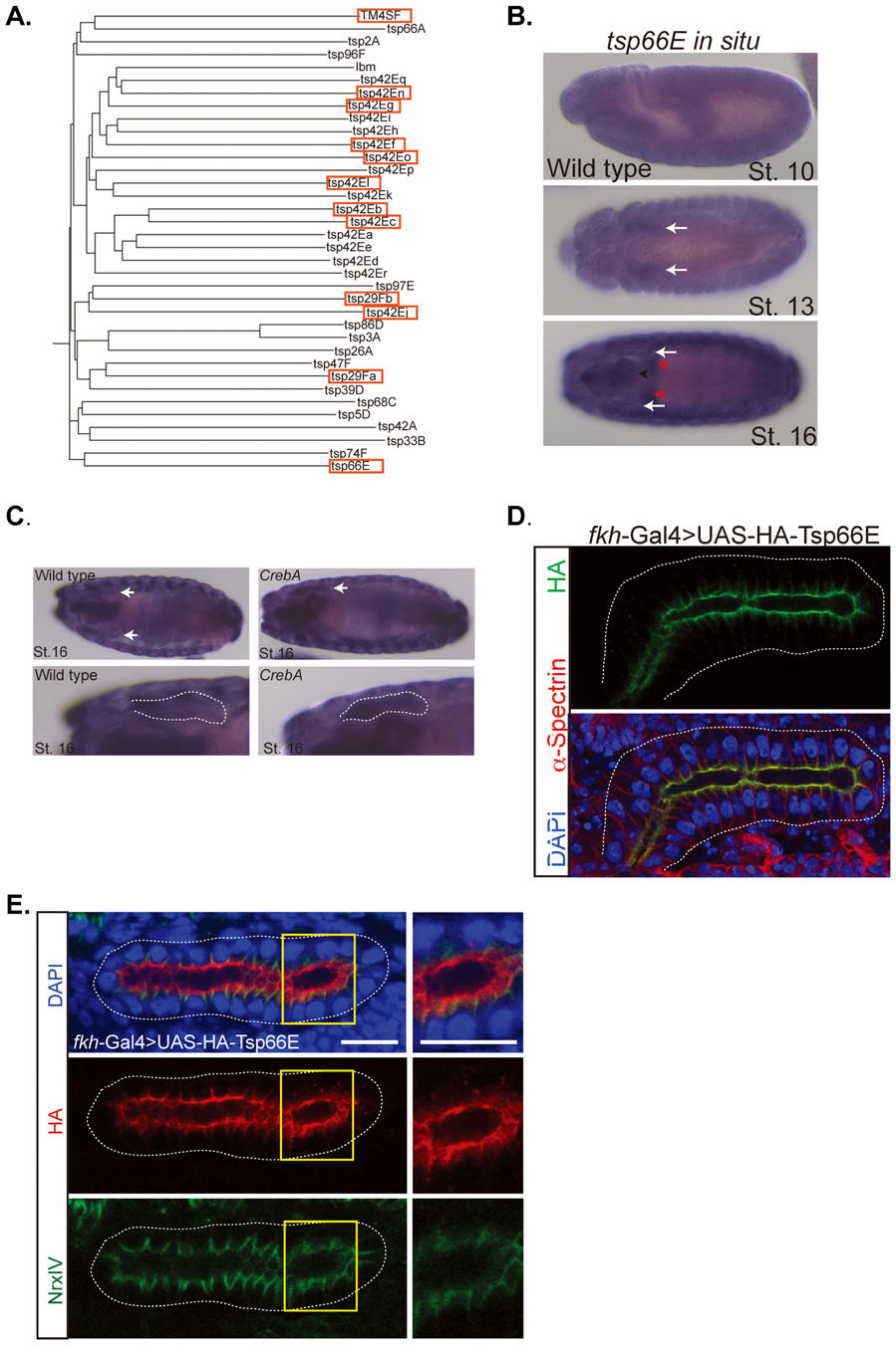
Tsp66E localizes to the apical surface and to SJs in embryonic SGs. (A) Phylogenetic tree of the 35 members of the tetraspanin superfamily in Drosophila. Red boxes indicate tetraspanins significantly upregulated in *CrebA* mutants, based on microarray data. (B) *In situ* hybridization of *tsp66E* in wild-type embryos at stages 10, 13 and 16. Arrows point to SG expression in stages 13 and 16. Red arrowheads denote the gastric caeca. (C) *In situ* hybridizations of stage 16 embryos with a *tsp66E* probe shows increased SG staining in the *CrebA* mutant compared to WT. Arrows point to SG in the upper panels and outline the SG in the lower panels. (D) *fkh*-Gal4 driven UAS-HA-Tsp66E localizes to the apical and lateral membranes in the salivary gland. The HA lateral membrane localization is confined to the area just below the apical surface. (E) SGs expressing UAS-HA-Tsp66E (red) and co-stained with the SJ marker NrxIV (green) reveal that the lateral localization of Tsp66E is at the SJ. Yellow boxes outline regions magnified in right panels. Scale bars: 5 µm.

To determine if other CrebA target genes contribute to the increased SJ-associated membrane, genes whose expression was significantly up or down in *CrebA* mutants, based on previously obtained microarray data, were examined (Fox et al., 2010; supplementary material Table S1). The most intriguing candidates emerged from among the upregulated genes, including a group of 12 tetraspanin genes. The Drosophila genome encodes 36 tetraspanin genes, of which almost half are found in a single cluster in cytological region 42D–E (Fig. 6A; Table 1). The unexpectedly high representation of members of this superfamily of plasma membrane-localized proteins in genes upregulated in CrebA mutants suggested a potential link to the excessive SJ membrane phenotype (Table 1). The tetraspanin genes whose expression goes up significantly in *CrebA* mutants are eight in the 42E cluster, two in cytological region 29F, one in cytological region 60, and one in cytological region 66, known as *Tsp66E*. In the embryo, *tsp66E* is expressed in multiple tubular organs. During stages 10–14, *tsp66E* is most highly expressed in the embryonic salivary glands (Fig. 6B). At stages 15 and 16, the SG staining becomes less prominent as the expression in additional organs, including the trachea, hindgut and epidermis becomes elevated. Importantly, this is the time frame when the SJs are forming in epithelial tissues. Consistent with the microarray data, *in situ* analysis reveals that *tsp66E* transcript levels remain at elevated levels in late stage *CrebA* mutant SGs (Fig. 6C). To localize Tsp66E protein, an HA-tagged version of Tsp66E that has been shown to rescue ovary defects associated with Tsp66E loss (Han et al., 2012) – UAS-HA-Tsp66E – was expressed in the SG using the *fkh*-Gal4 driver. Intense HA staining was observed along the apical surface of the SG, as well as somewhat less intense staining in a limited region of the lateral membrane, just below the apical surface (Fig. 6D). Co-staining embryos with NrxIV antisera revealed that the lateral HA-Tsp66E staining overlaps the SJ (Fig. 6E).

**Table 1.**
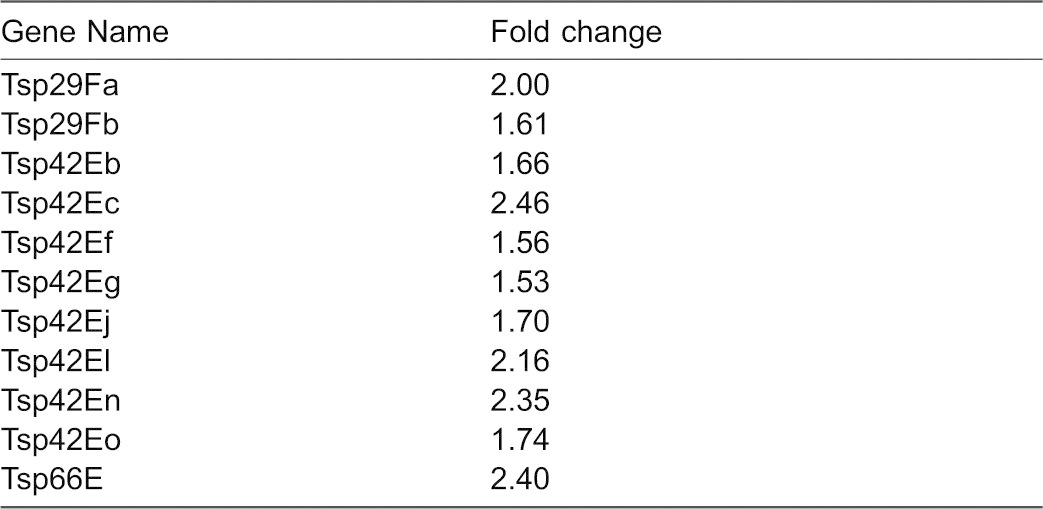
Tetraspanin family genes upregulated in CrebA mutants

**Fig. 7.**
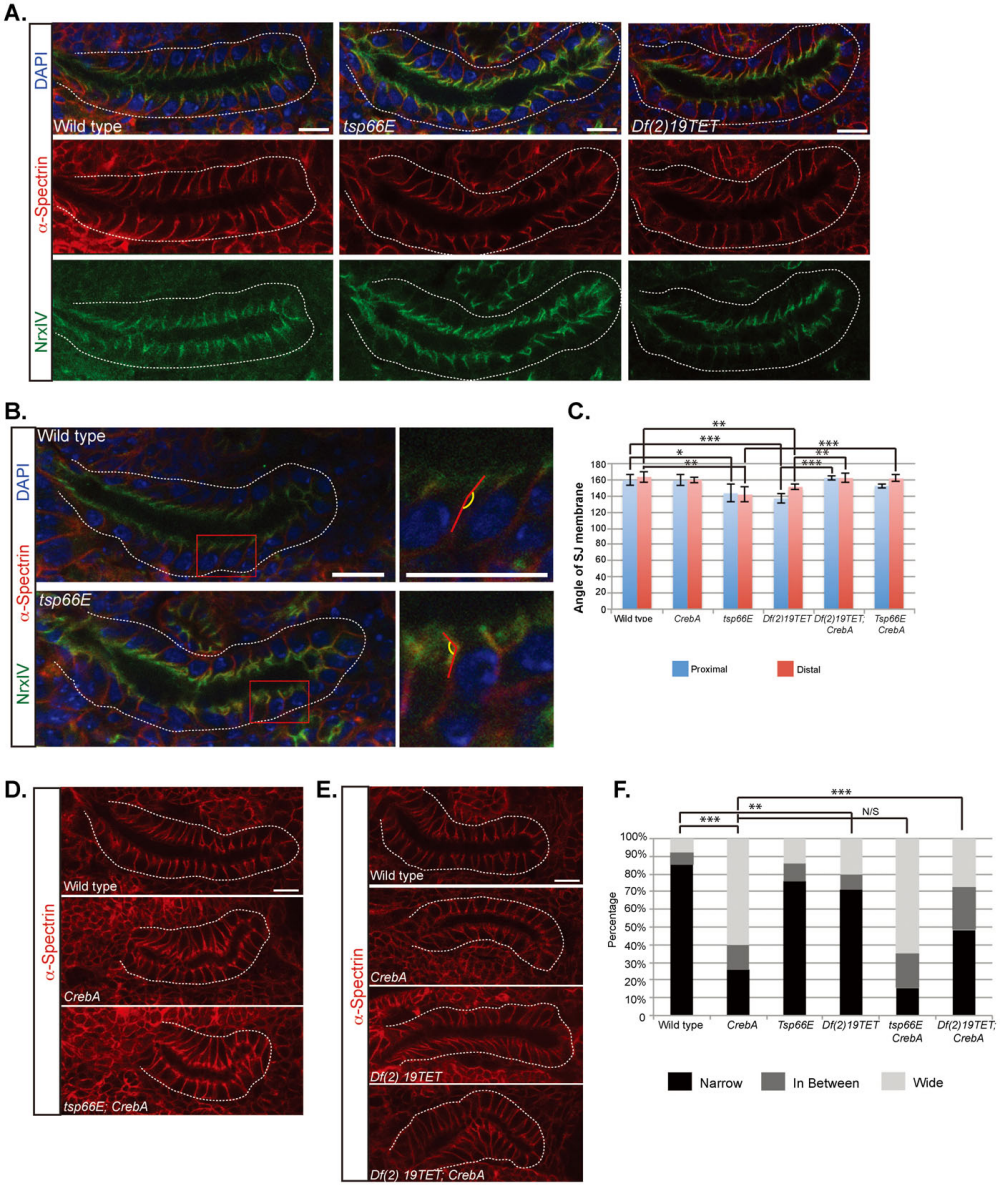
Tetraspanin loss results in irregularities in the SJ region of the membrane that are rescued by simultaneous loss of *CrebA*. (A) α–Spec (lateral membrane marker, red) and NrxIV (septate junction marker, green) staining reveal that *tsp66E* mutant and *Df(2)19TET* SGs show some “swaying” in the region of the SJ compared to the rest of the lateral membrane. Dashed line outlines the SGs. (B,C) Quantification of the angle between the SJ region and the remainder of the lateral membrane region reveals significantly more “sway” in the SJs of *tsp66E* and *Df(2)19TET* mutant SGs than in WT or *CrebA* mutants. Ten SJs at both the proximal (blue) and the distal (red) regions of ten individual SGs were analyzed (100 total SJs per genotype). Red boxes outline region magnified to the right. (D) α–Spec staining of wild type, *CrebA* and *tsp66E CrebA* mutant SGs. (E) α–Spec staining of wild type, *CrebA*, *Df(2)19TET*, and *Df(2)19TET; CrebA* mutant SGs. (F) At least 70 SJs from 3 individual glands of each genotype were classified as Narrow, wide or in between based on α-Spec staining. All SGs are embryonic stage 16. Error bars represent standard deviation. p-values were determined using the students t-test (C) or the g-test (F). * denotes p<0.1, ** denotes p<0.05, *** denotes p<0.01. Scale bars: 10 µm.

To ask if Tsp66E has a role in SJ formation or stabilization, *tsp66E^1^* mutants were obtained and the septate junction was examined using SJ markers. Whereas WT SJs are typically aligned perpendicular to the lumen, the SJ regions of *tsp66E* mutant SG cells appear to angle away from the rest of the lateral membrane, giving the cells a swayed appearance (Fig. 7A,B), a phenotype quantified by measuring the acute angle of sway relative to the rest of the lateral membrane (Fig. 7C). To test if loss of *tsp66E* could rescue the SJ membrane accumulation observed in *CrebA* mutants, we created embryos homozygous for null mutations in both genes and examined the SG lateral membranes. Although *tsp66E* loss did not rescue the extra membrane defect observed in *CrebA* mutant SGs (Fig. 7D), loss of *CrebA* rescued the SJ sway phenotype associated with *tsp66E* loss (Fig. 7C), perhaps due to the other tetraspanins whose expression is upregulated in *CrebA* mutants.

**Fig. 8.**
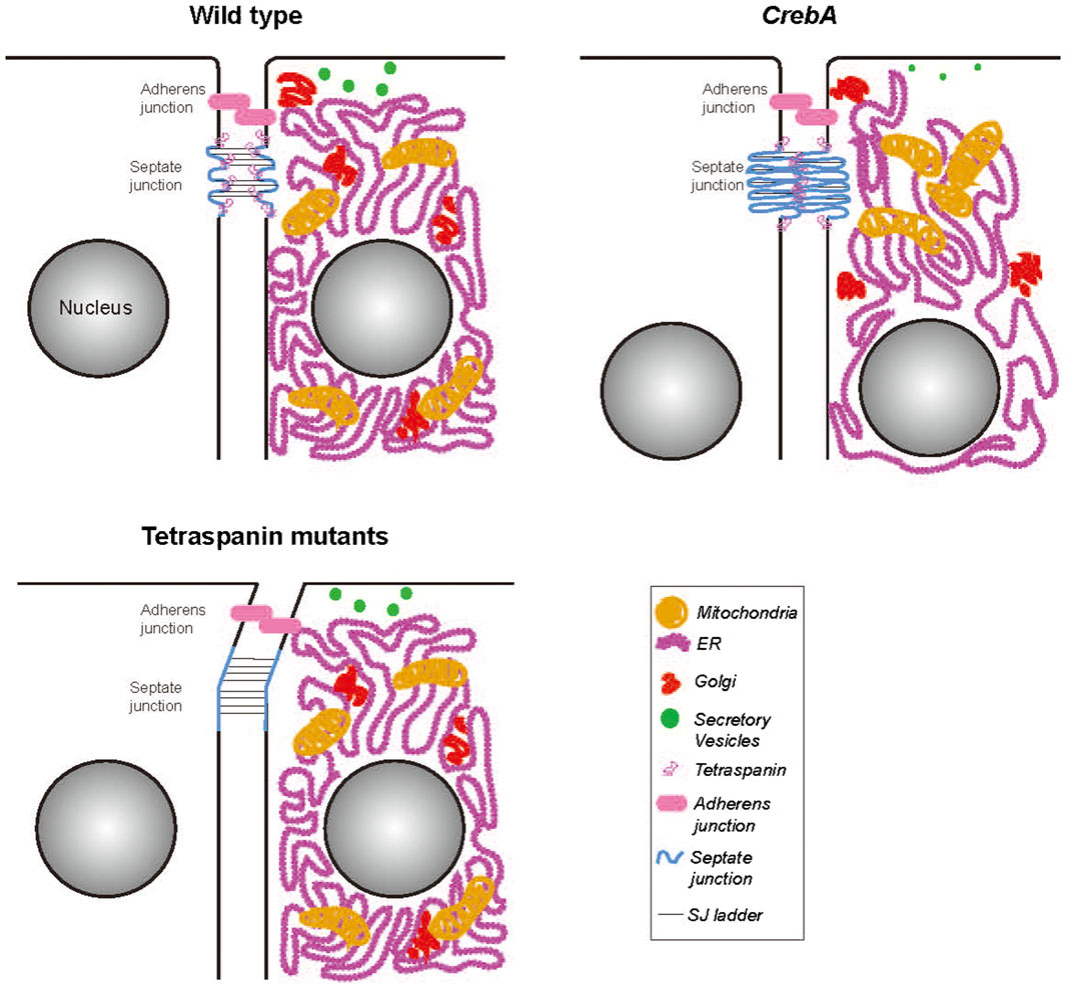
Cartoon representation of WT, *CrebA* and tetraspanin mutant cells with the differences in organelle localization and SJ structure highlighted. In WT SG epithelial cells, the SJs are slightly convoluted and there is an accumulation of tetraspanin proteins specifically at the SJ region. In *CrebA* mutants, there is a global reorganization of membranous organelles and the SJs become more convoluted, due to excess membrane. By microarray analysis, there is an increase in tetraspanin gene transcription presumably resulting in more tetraspanin protein at the SJ to stabilize the increased SJ membrane folds. In tetraspanin deficient cells, the SJs are slightly longer and less rigid, resulting in a swaying phenotype.

Tetraspanins are known to function in multi-protein complexes (Kovalenko et al., 2004; Kuhn et al., 2007; Nydegger et al., 2006; Yáñez-Mó et al., 2009), and individual tetraspanin proteins are typically not essential components of the complexes (Fradkin et al., 2002; Kovalenko et al., 2007). Due to these findings as well as the absence of mutations affecting any single Drosophila tetraspanin other than *Tsp66E*, the consequence of removing several tetraspanins was examined. *Df(2R)19TET* removes ten genes including nine tetraspanins in cytological region 42E; four of which were significantly upregulated in *CrebA* mutants (Table 1; Fig. 6A). Similar to the *tsp66E* loss-of-function mutant, the *Df(2R)19TET* deficiency also results in an SJ “swaying” phenotype, especially at the proximal end of the SG tube. We next made *Df(2R)19TET; CrebA* double mutants and stained SGs for α–Spec to see if the deficiency could rescue the *CrebA* mutant extra membrane phenotype and/or if loss of *CrebA* could rescue the SJ swaying defect observed with the deficiency. Indeed, we observed both a partial rescue of the *CrebA* excess SJ membrane defect as well as a rescue of the SJ swaying defect (Fig. 7E,F). These findings suggest that tetraspanins function as components of insect septate junctions and that their upregulation in *CrebA* mutants may contribute to the SJ targeting of excess membrane.

## Discussion

CrebA upregulates expression of genes encoding all known protein components of the early secretory pathway in multiple organs requiring increased secretory capacity (Abrams and Andrew, 2005; Fox et al., 2010). The timing and levels of CrebA expression in secretory organs corresponds to the level of secretory activity, with the salivary gland cells showing the highest levels of CrebA expression and highest levels of secretion per cell. Likewise, neuronal expression of CrebA is induced only later in larval stages, where it supports the increased expression of secretory components required for dendritic arborization (Iyer et al., 2013). Importantly, CrebA is not required for the basal levels of secretory activity occurring in most cell types and loss of CrebA primarily affects the cells that secrete the most, including the embryonic SG and epidermis. The SGs of *CrebA* mutants are crooked with an irregular lumenal matrix and the epidermal cells secrete a weakened, underdeveloped cuticle (Abrams and Andrew, 2005; Fox et al., 2010). At the cellular level, *CrebA* mutant SGs have fewer, smaller secretory vesicles and accumulate less secreted material in the lumen. Additional unexpected phenotypes include cellular relocalization of both secretory and non-secretory organelles and increased membrane accumulation at the septate junctions.

The repositioning of organelles observed in CrebA mutant SGs and epidermal cells appears to be linked to decreased secretory function since single mutations in individual components of the core secretory machinery result in a similar, albeit milder, organelle relocalization phenotypes. We propose that under conditions of reduced secretory capability, cells may concentrate the secretory machinery into smaller functional domains closer to the side of the cell with the highest secretory output – near the apical surface. We further propose that the relocalization of the secretory machinery indirectly affects the localization of “non-secretory” organelles, specifically nuclei and mitochondria. The apical repositioning of the mitochondria likely occurs through the physical linkage between the ER and mitochondria by the MAM – mitochondria-associated membrane – complexes, structures that tether the mitochondria to the ER for purposes of calcium and lipid exchange, as well as regulation of mitochondrial morphology, dynamics and function (Raturi and Simmen, 2013; Vance, 2014). The nuclei are likely repositioned to a more basal position simply due to the apical crowding by the secretory machinery and mitochondria.

We propose that the increased membrane observed in the SJs of *CrebA* mutant SGs is from the secretory organelles – specifically the ER and Golgi, both of which exhibit reduced staining of known protein components, with the ER also showing dramatic reduction in TEMs. Indeed, although CrebA regulates expression of genes encoding the protein components of the early secretory machinery, it does not affect expression of genes encoding the enzymes that synthesize or transport lipid membrane components (Fox et al., 2010). Thus, one would expect an excess of membrane due to the reduction of nearly all the secretory protein components that normally populate the ER and Golgi apparatus, especially in the SG, where CrebA and all of the protein components of the secretory machinery are most highly expressed. If the excess membrane remained associated with these organelles, the protein machinery would be significantly diluted, further diminishing secretory function. Thus, by concentrating the secretory organelle proteins into a smaller cellular domain and trafficking the excess lipid membrane to another cellular “repository”, *CrebA* mutant cells can maximize their limited secretory efficiency.

Regardless of the source of the excess membrane, why would the it accumulate at SJs and not some other cellular domain? Adding excess membrane to other subcellular organelles would effectively dilute the protein components, thus also compromising function. Similarly, trafficking excess membrane to either the apical or basolateral plasma membrane could affect cell shape, leading to distortions in overall organ shape, much like the phenotypes observed with loss or overexpression of proteins that control cell polarity. The SJ normally serves a fencing function, segregating apical lipids and proteins away from those in the basolateral domain. As cells (and consequently) organs change shape during development, having a membrane reservoir at the junction between polarized membrane domains should allow for efficient expansion of either the apical or basolateral membrane surface. Indeed, based on studies in which apical membrane components are overexpressed in the SG, the SJ domain also appears to be the most resistant to polarity conversion. Overexpression of apical membrane surface proteins, such as Cad99C and SAS, or the apical determinant Crb can confer apical character to the entire plasma membrane, with the exception of the SJ domain; the SJ domain is narrower, however, as if the membrane were stretched (Chung and Andrew, 2014). Thus, the SJ appears capable of absorbing extra membrane by folding it into more convoluted sheet-like structures when there is too much and stretching it out when membrane becomes limited (Fig. 8). In fact, the failure to carry out this function may contribute to the organ shape defects observed in the trachea with mutations in SJ proteins (whose localization to the SJ are interdependent); loss of SJ function results in significant apical surface expansion leading to convolutions and contortions of the entire tracheal tube. We propose that adding excess membrane to the SJ domain, which appears to function as a neutral membrane sink, minimizes the overall impact on organelle function, cell shape and polarity, all of which are important to secretory organ function.

We have identified the Tetraspanins as candidate mediators of the increased membrane packaging at the SJs (Fig. 7). Twelve of the thirty-six tetraspanins encoded in the Drosophila genome were significantly upregulated in *CrebA* mutant embryos. As their name implies, tetraspanins have four membrane spans with a very short cytosolic N-terminal domain and a short cytosolic C-terminal domain. Tetraspanins contain two extracellular loops, a more N-terminal shorter loop and a more C-terminal longer loop with at least four cysteines, which form disulfide bonds. In general, tetraspanins are thought to function as scaffolds that bring multiple proteins – including other tetraspanins, membrane-associated proteins, as well as extracellular and cytosolic proteins – into single membrane region, currently referred to as tetraspanin-enriched microdomains (Yáñez-Mó et al., 2009; Zhang and Huang, 2012). The relative abundance of tetraspanins in multiple cell types as well as their ability to associate with transmembrane receptors, potentially affecting the avidity of binding, make them especially good candidates as organizational units in the plasma membrane. Tetraspanins have been shown to associate with multiple proteins that localize to specific plasma membrane compartments, including the uroplakins (apical membrane), E-cadherin (adherens junctions), integrins (basolateral membrane), and claudins (vertebrate TJs and insect SJs) (Yáñez-Mó et al., 2009). The binding of individual tetraspanins to multiple membrane proteins, suggest that they may participate in different protein complexes depending on which partner proteins are also expressed. For example, Drosophila Tsp66E clearly localizes to distinct domains in different cell types. In the follicular epithelium of the Drosophila ovary, Tsp66E, the Drosophila orthologs of vertebrate KAI1/CD82, localizes to junctions near the basal surface where it affects actin polarity by regulating the localization of αPS2 integrin (Han et al., 2012). Loss of *tsp66E* in the follicular epithelium leads to defects in egg elongation and its loss enhances the wing blistering phenotypes observed with loss of αPS2 integrin (*inflated*). In the embryonic SG, Tsp66E localizes to the apical surface, extending into the lateral domain where it overlaps known SJ proteins. Loss of *tsp66E* in the SG affects SJ morphology, resulting in a swaying of the SJ region relative to the more basal regions of the lateral membrane. Similar SJ swaying defects are observed in the SGs with a deficiency removing nine distinct tetraspanins, suggesting that a subset of these molecules also contribute to SJ morphology. Finding that this deficiency partially rescues the excess membrane at the SJ observed in *CrebA* mutants is consistent with CrebA either directly or indirectly upregulating these proteins as a mechanism for packaging excess membrane in the most benign domain of the cell.

Interestingly, the vertebrate Tsp66E ortholog KAI1/CD82 is expressed in late-lineage oligodendrocytes and is hypothesized to restrict migration and promote differentiation of oligodendrocytes, the cells that myelinate CNS neurons (Mela and Goldman, 2009). Importantly, vertebrate TJs are found between the myelin sheaths of the extended plasma membrane of oligodendrocytes, an observation made more than 40 years ago (Bronstein and Tiwari-Woodruff, 2006), suggesting that vertebrate TJs (the radial component of myelinated axons) are the sites where the membrane components of myelin sheaths are organized (Dermietzel and Kroczek, 1980). Similarly, ladder-like SJs form between the outer and inner glial cell membranes that provide the same insulating function as the myelinating oligodendrocytes and Schwann cells of vertebrates (Banerjee and Bhat, 2008; Banerjee et al., 2006; Blauth et al., 2010). Thus, a clear precedent exists for adding membrane to vertebrate TJs and insect SJs during normal development. TJs and SJs are located at the cellular domains that undergo elaborate expansion of the plasma membrane in the specialized cells that ensheath neurons and their axons – the oligodendrocytes, Schwann cells and glia. Our findings suggest that SJs (and potentially vertebrate TJs) provide a plasma membrane reservoir in multiple cell types, including in the polarized epithelia of the Drosophila salivary gland.

## MATERIALS AND METHODS

### Fly strains

The *CrebA^wR23^* protein null allele was used for all experiments and is referred to as the *CrebA* mutant throughout the text (Andrew et al., 1997). The following lines were obtained from the Bloomington Stock Center: UAS-mito-GFP.AP/CyO, UASp-GFP.Golgi, UAS-ER-YFP, and UAS-Grasp65-GFP. The secretory pathway mutant alleles were also obtained from Bloomington and included: SrpRβ (rk561), γCop (kg06383), Spase12 (EY10774), δCop (g0051), Sec63 (EY04730), Sec13 (01031), all of which had P-element insertions in the open reading frame, Sec61γ (EP1511) and Sar1 (05712), which had insertions in the first exon, and Sec61β (07214), which had a P-element insertion just upstream of the first exon. Other lines used in this work included: *fork head* (*fkh*)-Gal4 (Henderson et al., 1999), UAS-MannII-GFP (Velasco et al., 1993), UAS-CAAX-GFP (referred to as UAS-TMEM-GFP), UAS-PLCδ-GFP (von Stein et al., 2005), *Df(2)19TET* (Fradkin et al., 2002), UAS-HA-Tsp66E and *tsp66E^1^* (Han et al., 2012).

All lethal mutations were maintained over balancer chromosomes containing either a lacZ or GFP transgene to allow for unambiguous identification of homozygous mutant embryos. Mutant embryos were compared to both wild-type embryos and to their heterozygous siblings, which were indistinguishable. Unless noted, the genotype of the wild-type controls shown for all images is Oregon R.

### Antibody staining

Embryo fixation and immunohistochemistry were performed as previously described (Reuter et al., 1990) with the exception of the phalloidin and tubulin staining. For phalloidin staining, embryos were fixed in 1:1 formaldehyde-saturated heptane and the vitelline membranes were removed manually. Embryos were then incubated in PBT with rhodamine-conjugated Phalloidin at a concentration of 1:500. For tubulin staining, embryos were fixed in methanol, washed with PBSTB and then incubated with Alexa-488 conjugated α–Tubulin antibody (Invitrogen) at a concentration of 1:500. The antibodies and concentrations used for this study are as follows: mouse α-Crb (1:100, Developmental Studies Hybridoma Bank (DSHB)), rabbit α-SAS (1:500, D. Cavener, Pennsylvania State University, PA), mouse α–βgal (1:500, Promega), mouse α–α–Spec (1:2, DSHB), mouse α-2A12 (1:10, DSHB), guinea pig α-Cora (1:2000, R. Fehon, University of Chicago, Illinois), rabbit α-NrxIV (1:2000, H. Bellen, Baylor University, TX), α-Dlg (1:500, DSHB), α-GFP (1:10,000, Molecular Probes). Fluorescently-tagged secondary antibodies (Alexa-488, Alexa-555, Alexa-568 or Alexa-647, Molecular Probes) were used at a dilution of 1:500. Imaging was performed on a Zeiss LSM 510 Meta laser-scanning confocal microscope equipped with Zen software or a Zeiss 700 laser-scanning confocal microscope also equipped with Zen software.

### *In situ* hybridization

*In situ* hybridization was performed as previously described (Lehmann and Tautz, 1994). Antisense RNA probes were directed to the first exon of the Tsp66E coding region.

### Transmission electron microscopy

Wild type (Oregon R) and *CrebA* mutant embryos were processed for electron microscopy as described previously (Fox et al., 2010). At least three individual SGs were examined for each genotype. Images were obtained on a Phillips EM120 transmission electron microscope.

### Quantification of mutant phenotypes

#### SJ angle measurements

Images stained with α–Spec (red) and NrxIV (green) were processed using ImageJ software. Acute angle measurements were obtained using the angle function in which the acute angle was measured between two lines. The first line was taken along the lateral membrane and then the second line was generated from the position where the NrxIV staining meets the α–Spec staining (Fig. 6B). Measurements were taken for ten individual cells in both the proximal and the distal portion of each gland. At least three glands were analyzed per genotype. P-values were generated using the Student's t-test.

#### SJ size measurements

In wild type embryos staining for the SJ is a thin, almost straight line; in *CrebA* mutants, the SJ region is expanded. SJs were counted and categorized based on thickness, either narrow, wide or somewhere in between from at least 70 cells from three salivary glands stained with α–Spec for each genotype. Significance was determined using a G-test.

## Supplementary Material

Supplementary Material
