# Supplementary material for: Changes in organelle position and epithelial architecture associated with loss of CrebA
Source: Biol Open. 2015 Feb 13;4(3):317–30. doi: 10.1242/bio.201411205 (PMC4359738; doi:10.1242/bio.201411205)
Supplement: Supplementary Material [file supp_4_3_317__index.html]

Changes in organelle position and epithelial architecture associated with loss of CrebA — Supplementary Material 

# Changes in organelle position and epithelial architecture associated with loss of CrebA

## bio.201411205

**Files in this Data Supplement:**

- Table S1 - List of all genes whose expression went up by at least 1.5 fold in CrebA mutant embryos relative to wild type embryos (p<0.05).
